# Women’s experiences following emergency Peripartum hysterectomy at St. Francis hospital Nsambya. A qualitative study

**DOI:** 10.1186/s12884-020-03428-3

**Published:** 2020-11-25

**Authors:** Patrick Pilli, Peter Sekweyama, Anthony Kayira

**Affiliations:** 1 Department of Obstetrics and Gynecology, St. Francis Hospital Nsambya, P.O.Box 7146, Kampala, Uganda; 2 Mother Kevin Postgraduate Medical School, Uganda Martyrs University, P.O.Box 7146, Kampala, Uganda

**Keywords:** Emergency peripartum hysterectomy, Emotional experiences after traumatic birth, Severe postpartum hemorrhage

## Abstract

**Background:**

Emergency peripartum hysterectomy (EPH) is a known remedy for saving women’s lives when faced with the challenging situation of severe post partum hemorrhage not responsive to conservative management. However, EPH by its nature is also a traumatic birth event that causes serious physical, emotional and psychological harm. Unfortunately at St. Francis Hospital Nsambya nothing much is known about these experiences since no study has been undertaken and these women are not routinely followed up. The purpose of this study was to explore these emotional experiences.

**Methods:**

This was a qualitative phenomenological study carried out between August and December 2018. All those women who had undergone EPH between January 2015 and August 2018 were eligible to participate in the study. Purposive sampling was used. 18 women were interviewed before saturation was reached. All interviews were audio-recorded and then transcribed verbatim. Thematic analysis was used to analyze the data.

**Results:**

Three major themes were identified as the main experiences of these women in this study and they were; Loss of Womanhood, Joy for being alive and Loss of marital safety.

**Conclusion:**

Women experience serious emotional consequences following EPH. We recommend routine follow-up to help appreciate these experiences and advise them on appropriate mitigating measures.

## Background

Emergency peripartum hysterectomy (EPH) is the surgical removal of the uterus within 24 h of delivery due to severe post partum hemorrhage not responsive to conservative management. It is a known remedy for saving women’s lives [1–3]. EPH however is also a traumatic birth event which causes serious physical, emotional, spiritual and psychological harm [4, 5].. Studies have found that when these negative emotions are not fully explored and addressed, most of these mothers end up with depression, having low quality of life and loss of self worth [6, 7]. Mbekenga et al. (2011) found out that every woman primarily comes to the delivery room to experience joy, elation and the pride that comes with mothering. None of them come expecting to lose a uterus [8–10]. So the sudden loss of their uterus under such catastrophic circumstances is traumatic and associated with negative emotional experiences. Among these are a sense of worthlessness, sadness, emptiness and total loss [4, 5, 11]. Further more due to the permanent infertility that follows hysterectomy, it causes these women to be divorced or abandoned by their husbands, subjected to extreme forms of gender based violence and other times ostracized from society [5, 12].

How ever some studies have found out that a few of these women feel a sense of gratitude for being alive despite the near death experience [13–15].

Globally 1 in 1000 women will lose their uterus during child birth through EPH [1, 16]. This is mainly due to severe post partum hemorrhage secondary to abnormal placentation (placenta previa or placenta accreta), abruption placenta, uterine atony, ruptured uterus and genital tears [17–19] In Sub-Saharan Africa, the incidence of EPH is five times higher than the global average mainly because of high parity, lack of skilled attendance at birth, prolonged and obstructed labor [14, 20–22]. At St. Francis Hospital Nsambya, the prevalence of EPH during the study period was 1.2/1000 births (Hospital records 2015–2018) however the emotional experiences of these women is unknown since no routine follow-up or psychotherapy sessions are conducted. Anecdotal evidence indicate that these women are being subjected to significant family ridicule, physical violence and sometimes outright divorce because of not being able to give birth to more children. At St. Francis Hospital Nsambya much as EPH is being done, no study has been undertaken to explore these experiences and no routine follow-up is being done. The purpose of this study therefore was to explore womens’s experiencing following EPH at St. Francis Hospital Nsambya.

## Methods

### Study design

We employed a phenomenological qualitative research design. Phenomenology is the study of people’s lived experiences or how people experience things in their conscience or the study of the character and the appearance of peoples experiences in their minds [23, 24]. We employed this design because we wanted to understand the lived experiences of these women following the EPH.

### Study area

This study was carried out at a referral teaching hospital, St. Francis Hospital Nsambya, a Catholic founded private not for profit (PNFP) hospital located 3.6 km south of Kampala city centre in Makindye Division.

### Sampling process

The study participants were women who had undergone EPH at the hospital between January 2015 and August 2018. Purposive sampling was used. We began by extracting the records of these women from the major operating theatre books. A total of 64 women were identified. Based on the inclusion criteria of the participant should be 18 years and above, live within a 50 km radius of the hospital and should have given birth at St. Francis Hospital Nsambya. 38 of them met these criteria. The principal investigator then phoned each of these 38 potential participants and explained to them the purpose of study, how it will be conducted and also sought for their consent to come back to the hospital for a face to face interview that would be audio recorded. 22 verbally consented. A tentative time and date for the interview was also agreed upon. Reminder phone calls were made at 72 h and 24 h prior to the face to face interview to confirm the appointment or to reschedule incase of any changes. On the day of the scheduled interview each participants was picked up from the main hospital gate by a member of the study team and led to a private room within the hospital away from the labour wards in order to minimize flash backs and bad memories. A nurse counselor was at hand to provide counseling incase the need arose. Before the interview commenced a formal written consent was obtained. Each interview lasted about 45 to 60 min and they were conducted by a trained research assistant in the presence of the principal investigator whose role was to mainly ask probing questions. A third member of the team was also present to take notes for the purpose of capturing the non verbal cues. Out of the 22 participants 18 were interviewed before saturation was reached. The remaining four participants were appreciated for their willingness to participate, informed that the required information was obtained and the study findings would eventually be shared with them.

### Interviews

We used an interview guide. This guide was first piloted on women who had undergone traumatic deliveries and approved by a social scientist specialized in phenomenological studies. The guide had a section for demographic characteristics, open ended questions and probes.

### Data analysis

We used thematic analysis. After completing the first five interviews the research assistants transcribed them verbatim. A copy of the transcripts and the audio recordings were then made available to each of the three investigators. They each individually read through the transcripts and listened to the recordings several times to come up with meaning units. Subsequent transcripts were analyzed while taking into consideration these first set of meaning units unless new ones emerged. These meaning units were then coded together to form categories and from the different categories the three themes emerged.

### Ethical considerations

Ethical approval was received from the Research and Ethics Committee of St. Francis Hospital Nsambya (REC No: UG-REC-020). A voluntary written informed consent was obtained from all participants and participant numbers were used as identifiers instead of their names for confidentiality purpose.

## Results

18 women were interviewed before saturation was reached. Their average age at the time of hysterectomy was 34 years. All participants had a minimum of secondary education. Uterine atony was the commonest risk factor leading to EPH followed by placental abnormalities. All except one were multiparous and caesarian section delivery was more prevalent than vaginal delivery. Majority of the participants were ethnic Baganda and Christians (Table 1).

**Table 1:** Socio-demographic Characteristics

Participant No.	Age group (Yrs	Parity	Delivery Mode	Indication for EPH	Education level	Religion	Marital status	Living children
1	25–30	2	SVD	Uterine atony	Degree	Moslem	Married	2
2	30–35	3	C/S	Uterine atony	Degree	Catholic	Married	3
3	30–35	3	C/S	Placenta accrete	Diploma	Catholic	Married	3
4	30–35	4	C/S	Abruption placenta	Degree	Protestant	Married	4
5	25–30	3	SVD	Cervical tear	Degree	Protestant	Married	2
6	45–50	10	C/S	Ruptured uterus	Secondary	Protestant	Widowed	8
7	30–35	1	C/S	Uterine atony	Diploma	Catholic	Divorced	1
8	35–40	6	C/S	Placenta previa	Secondary	Born again	Married	5
9	20–25	2	C/S	Uterine Atony	Secondary	Catholic	Married	2
10	35–40	5	C/S	Placenta Previa	Secondary	Catholic	Married	4
11	25–30	3	C/S	Ruptured uterus	Secondary	Moslem	Separated	2
12	30–35	3	C/S	Uterine atony	Degree	Catholic	Separated	3
13	35–40	4	SVD	Ruptured uterus	Degree	Catholic	Married	3
14	35–40	4	C/S	Ruptured uterus	Diploma	Catholic	Married	3
15	35–40	3	C/S	Placenta accrete	Secondary	Protestant	Married	3
16	35–40	3	C/S	Uterine Atony	Degree	Catholic	Married	3
17	35–40	6	C/S	Uterine Atony	Secondary	Born again	Separated	6
18	40–45	3	C/S	Uterine Atony	Degree	Catholic	Married	3

*SVD* Spontaneous vaginal delivery, *C/S* Caesarian section.

### Women’s experiences following EPH

The experiences of these women were summarized into three major themes and they were they were; Loss of womanhood, Joy for being alive and Loss of marital safety.

### Loss of womanhood

Most of the women believed that womanhood was defined by the presence of a uterus. They felt deeply that having no uterus was equivalent to being less of woman, worthless, manly and dismembered. They wondered how one can be a complete woman without getting monthly periods. Some said they now feel a deep hole inside their tummies; others described their feelings as emptiness, incompleteness and loss.

*“I felt sad, lost and empty … I wanted five children of my own but I only have two now, being a woman has now lost meaning …*” *Participant No.2.*

*“I am an only child, I wanted to have a big family that’s why I decided to start producing at an early age, now look at what has happened hmmm, i can no longer achieve my goal so being a woman is now meaningless” Participant No.1.*

Others reported experiencing loss of feminine attributes such as attractiveness, beauty and softness following the EPH. Some reported reduction in sexual desire, vaginal dryness and the inability to sexually satisfy their husbands.

*“Following the news that my uterus was removed I felt very unattractive to my husband, I lost confidence in be. My husband also felt I was no longer woman enough so we ended up divorcing” Participant No.7.*

*“After my first operation I was determined to push the next baby. The doctors said my bones would not allow but I insisted. I wanted to be like other women … unfortunately I got complications which led to me losing my uterus, now I feel less of a woman”. Participant No.9.*

### Joy for being alive

Women who were unconscious by the time of being taken to theatre and those who didn’t know how the ended up in theatre felt a great sense of joy for being alive. They said the mere fact that they are still alive and breathing was a miracle. Seeing their families again after being in coma was the happiest moment of their lives described some. Another group of women who felt total relief were those who conceived accidentally while on contraceptives. These women said deep inside them, they knew they had completed their families so the pregnancy was a mistake in the first place. So knowing that they are never going to give birth again was the best news to their ears. Almost all of them agreed that life was more important than the uterus.

*“I knew it was over for me because as they were rolling me to theatre I was seeing “angels” and my relatives who had died long time ago… being alive today is still a miracle to me, am so happy to God saving my life” Participant No.13.*

*“I was giving birth to my tenth child, so loosing the uterus was not an issue to me, in any case am happy because it has saved me from having more children which I never wanted in the first place” Participant No.6.*

Some believed God had given them a second chance to live again; they were told that colleagues who went through the same ordeal never made it so to them this was a divine intervention from God.

*“Initially I was very depressed, stopped working and kept away from people. One day my good friend came to visit me and she told me to count myself lucky for being alive because two of her other friends didn’t make it after going through such a similar operation … from that day forward she said her life changed, she learnt to appreciate life and be grateful God” Participant No.3.*

### Loss of marital safety

Most of the women felt so vulnerable and at the mercy of their husbands, for the first time in their marital lives they felt unsafe and worthless. The majority were not sure what their husbands were thinking or planning because most of them didn’t want to talk about it. Not being able to give birth in this part of the world is like a curse because most marriages are sustained by the woman’s ability to bear children. The fear of abandonment was overwhelming. Some men openly told these women to vacate their homes; others went on to get new mistresses.

*“We separated one year after the operation, my husband would beat and abuse me saying I was an empty tin …” Participant No.12.*

*“My husband abandoned our home, he was busy running after young girls, he rarely gave me money …” Participant No.14.*

## Discussion

We found Loss of womanhood, Joy for being alive and Loss of marital safety as being the major experiences of women following EPH at St. Francis Hospital Nsambya. Most of these women considered the uterus to be their most important organ of femininity. It defined their womanhood and therefore its absence was a major blow their identity. Womanhood also means being able to have monthly periods unfortunately EPH deprived them of this women specific physiological process. This left many questioning their womanhood. The loss of the uterus therefore virtually affected every aspect of the women’s life ranging from their body image, confidence, sexual relationship, work place relationships and their social standing in the society. Majority felt inferior to their peers, less deserving of their husbands and worthless to the community. According to Elmir et al. a woman’s definition of her womanhood or its lack of affects almost every aspect of her life including her confidence, relationship with her husband and her work life [5]. According to De la Cruz et al. those women who didn’t felt less womanly after EPH were at a higher risk of developing a major depression [4]. However the loss of a uterus was a blessing in disguise to some women especially those who never wanted more children and are against modern contraceptive methods. EPH gave them the opportunity to now focus on raising their children and streamlining their careers. These women were also grateful to God for sparing their lives. They thought that they would never see their families again. Being alive was a miracle to many and so they felt joy for being alive. Those who used to never appreciate life now treasured it profoundly; they described this maternal near miss situation as a turning point in their lives. They believed God had spared their lives for reason, so they are going to devote the rest of their life to serving him and humanity. Mbekenga, C. K., et al. found that women were extremely happy for having survived through the uncertainties of labour and delivery.

Much as the women were happy for being alive, the reality of permanent infertility quickly set and they realized it was never going to go away easily. The fear for the safety of their marriage of their marriage was so palpable. This is because in most Ugandan societies marriage equals to children, the current fertility rate as of 2019 was 5.4 children per woman [25] so not being able to have more children contributes significantly to the future of the marriage. Because of their inability to have more children these women reported being subjected to all forms of gender based violence such physical abuse, emotional abuse, deprivation of resources and sexual violence. They reported being abandoned by their spouses, thrown out of their matrimonial homes and forcefully separated from their children. Marital safety was a significant finding in this study. Most women confessed that the being fertile and having children was the primary for reason receiving financial support from their husbands. This safety net was now in great jeopardy because of the loss of the uterus and the idea of raising children alone was untenable to many. Most of the men ended up remarrying or having extramarital affairs all in the guise of wanting more children. De la Cruz et al. found numbness, hopelessness and uncertainty about the marital future following the loss of a uterus [4].

The women reported that regular follow-ups, counseling, joining women groups dealing with permanent disability and having a supportive husband contributed greatly to helping them cope with their loss.

### Limitations

The findings of this study were limited to women who had EPH in an urban and a peri-urban setting. 80% of the women were ethnic Baganda so the findings might not be applicable in other parts of Uganda.

## Conclusion

In conclusion women experience severe emotional consequences following EPH. The study found Loss of womanhood, Joy for being Alive and Loss of marital safety as the major themes following EPH. We recommend regular follow-ups of these women so as to help appreciate these experiences and advise on appropriate mitigating measures.

## Supplementary Information

**Additional file 1: Supplementary file 1** Interview guide.

**Additional file 2: Supplementary file 2** point by point response to editor and reviewers comments.

## Data Availability

The data sets generated and analyzed during this study are available from the corresponding author on reasonable request.
