# Supplementary material for: Women’s experiences following emergency Peripartum hysterectomy at St. Francis hospital Nsambya. A qualitative study
Source: BMC Pregnancy Childbirth. 2020 Nov 25;20:729. doi: 10.1186/s12884-020-03428-3 (PMC7687817; doi:10.1186/s12884-020-03428-3)
Supplement: Supplementary file 1 — Additional file 1: Supplementary file 1 Interview guide. [file 12884_2020_3428_MOESM1_ESM.docx]

**IN-DEPTH INTERVIEW GUIDE**

1. **Tell us about that pregnancy in general**

**Probes:**

- was ANC attended
- was there any discussion about the possibility of losing your uterus

1. **Describe how you felt following the lose of the uterus**

**Probes:**

- Immediately after the operation
- As time passed by

1. **Anything else you would like to add?**

**Thank you for your time**
