# Supplementary material for: Women’s experiences following emergency Peripartum hysterectomy at St. Francis hospital Nsambya. A qualitative study
Source: BMC Pregnancy Childbirth. 2020 Nov 25;20:729. doi: 10.1186/s12884-020-03428-3 (PMC7687817; doi:10.1186/s12884-020-03428-3)
Supplement: Supplementary file 2 — Additional file 2: Supplementary file 2 point by point response to editor and reviewers comments. [file 12884_2020_3428_MOESM2_ESM.docx]

**EDITORS COMMENTS**

- Under ethical considerations: Yes a voluntary informed consent was obtained from each participants
- The English version of the interview guide has been uploaded as a supplementary file
- Potential identifier especially occupation of participants has been omitted.

**REVIEWERS COMMENTS)**

- Background: line 34-36, the word loose has been corrected to mean lose.
- Background: At St. Francis Hospital Nsambya much as EPH is being done, no study has been undertaken to explore these experiences and no routine follow-up is being done. The purpose of this study therefore was to explore womens’s experiencing following EPH at St. Francis Hospital Nsambya. This has been moved to the final paragraph of the background as recommended.
- Methods section: principle investigator has been changed to principal investigator through out.
